# Catatonia Secondary to Sudden Clozapine Withdrawal: A Case with Three Repeated Episodes and a Literature Review

**DOI:** 10.1155/2017/2402731

**Published:** 2017-03-15

**Authors:** John Bilbily, Betsy McCollum, Jose de Leon

**Affiliations:** ^1^College of Medicine, University of Kentucky, Lexington, KY 40506, USA; ^2^Pharmacy, Eastern State Hospital, Lexington, KY 40511, USA; ^3^University of Kentucky Mental Health Research Center, Eastern State Hospital, Lexington, KY 40511, USA; ^4^Psychiatry and Neurosciences Research Group (CTS-549), Institute of Neurosciences, University of Granada, 18971 Granada, Spain; ^5^Biomedical Research Centre in Mental Health Net (CIBERSAM), Santiago Apóstol Hospital, University of the Basque Country, 01004 Vitoria-Gasteiz, Spain

## Abstract

A literature search identified 9 previously published cases that were considered as possible cases of catatonia secondary to sudden clozapine withdrawal. Two of these 9 cases did not provide enough information to make a diagnosis of catatonia according to the Diagnostic and Statistical Manual, 5th Edition (DSM-5). The Liverpool Adverse Drug Reaction (ADR) Causality Scale was modified to assess ADRs secondary to drug withdrawal. From the 7 published cases which met DSM-5 catatonia criteria, using the modified scale, we established that 3 were definitive and 4 were probable cases of catatonia secondary to clozapine withdrawal. A new definitive case is described with three catatonic episodes which (1) occurred after sudden discontinuation of clozapine in the context of decades of follow-up, (2) had ≥3 of 12 DSM-5 catatonic symptoms and serum creatinine kinase elevation, and (3) required medical hospitalization and intravenous fluids. Clozapine may be a gamma-aminobutyric acid (GABA) receptor agonist; sudden clozapine withdrawal may explain a sudden decrease in GABA activity that may contribute to the development of catatonic symptoms in vulnerable patients. Based on the limited information from these cases, the pharmacological treatment for catatonia secondary to sudden clozapine withdrawal can include benzodiazepines and/or restarting clozapine.

## 1. Introduction

The literature has clearly established that antipsychotics can cause catatonia [1, 2]. Catatonia cases secondary to the intake of both first-generation [1–3] and second-generation antipsychotics [2, 4] have been described. There may be a spectrum between antipsychotic-induced catatonia and neuroleptic malignant syndrome (NMS) [5–7]. The incidence of NMS has been reduced after the introduction of second-generation antipsychotics [7]. A differential characteristic from clozapine is that it has been associated with NMS only on rare occasions. In a review of 68 published NMS cases associated with second-generation antipsychotics, there were 21 clozapine cases [8]. Most of the NMS cases associated with clozapine demonstrated atypical presentations [8, 9]. In spite of these cases, clozapine may be the best antipsychotic for rechallenging patients who developed NMS with other antipsychotics [10]. Manu et al. [11] reviewed the cases of five clozapine-induced NMS patients who were rechallenged with clozapine using slow titrations and creatinine kinase (CK) monitoring; all five rechallenges were successful and did not result in NMS.

**Figure 1:**
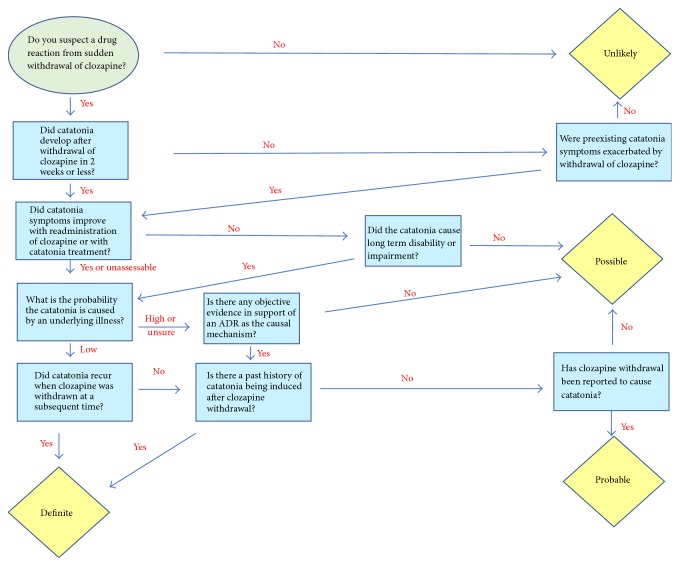
Modification of the Liverpool Adverse Drug Reaction Causality Assessment Tool [25] to retrospectively assess the likelihood that clozapine withdrawal caused the catatonia episode.

Another characteristic which makes clozapine appear different from the majority of antipsychotics is that abrupt clozapine discontinuation is frequently associated with withdrawal symptoms [12]. Clozapine is characterized by blocking multiple receptors including the dopamine, muscarinic, serotonergic, histaminic, and *α*_1_ adrenergic receptors. In a review on clozapine withdrawal, Verghese et al. [12] proposed that time course and clinical features of clozapine withdrawal may be explained by cholinergic overdrive and gamma-aminobutyric acid (GABA) supersensitivity. After clozapine withdrawal, some individuals have symptoms suggestive of a cholinergic rebound including nausea, vomiting, diarrhea, headache, agitation, confusion, and diaphoresis. These symptoms are probably explained by the antimuscarinic properties of clozapine and appear to respond to anticholinergic treatment [13]. Other individuals appear to have worsening psychosis and/or abnormal movements [14]. More importantly, in very rare instances, clozapine withdrawal has been associated with catatonic symptoms [15–24].  Table 1 describes the 9 cases that we identified in a PubMed search (Footnote 1). Catatonia episodes were rediagnosed using the Diagnostic and Statistical Manual, 5th Edition (DSM-5). The Liverpool Adverse Drug Reaction Causality Assessment Tool (LADRCAT) is used to assess adverse drug reaction (ADR) causality [25]. We modified it to retrospectively assess ADRs secondary to drug withdrawal. Then, we used the Modified LADRCAT (MLADRCAT) (Figure 1) to establish the likelihood that clozapine withdrawal caused the catatonia episodes in prior published cases (last column in Table 1) and in our patient who developed three catatonic episodes after three instances of sudden clozapine withdrawal.

## 2. Case Presentation

### 2.1. Assessments

#### 2.1.1. Assessments Using DSM-5 Catatonia Criteria

The DSM-5 Criterion A requires having ≥3 of the 12 listed catatonic symptoms needed to make the diagnosis. After reviewing all available information, the first and last authors discussed and agreed on the presence of DSM-5 Criterion A in our patient and in 7 of 9 of the previously published cases.

#### 2.1.2. The MLADRCAT

The LADRCAT [25] allows the diagnosis of an adverse drug reaction (ADR) while a drug is used for treatment, but it provides no system for diagnosing an ADR after drug withdrawal. To retrospectively assess the likelihood that clozapine withdrawal caused the catatonia episode, we modified the scale (MLADRCAT). This modified scale established the probability that clozapine withdrawal explained the three catatonic episodes of our patient (Figures  S10–S12 in Supplementary Material available online at https://doi.org/10.1155/2017/2402731) and the catatonic episodes of previously published cases (Figures  S1–S9).

#### 2.1.3. Laboratory Studies

The commercial laboratory tests routinely used by the psychiatric state hospital were employed to measure trough steady-state serum clozapine and norclozapine concentrations in ng/mL. The mean ± standard deviation (SD) for serum concentrations is described for clozapine, norclozapine, and the total (adding both). The recommended therapeutic range for trough serum clozapine concentration is 350–600 ng/mL [26]. Serum CK levels (normal laboratory range: 21–232 IU/L) were used as an objective measure of catatonia severity [27].

### 2.2. Prior History

#### 2.2.1. Description of the Patient

**Figure 2:**
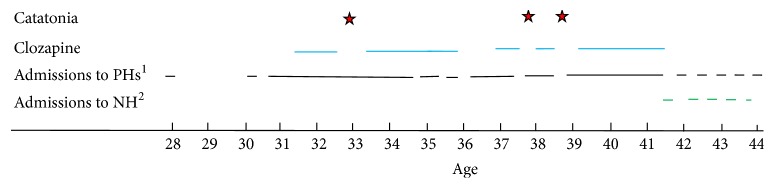
Longitudinal course of our patient including catatonic episodes, clozapine trials, and admissions to psychiatric facilities. ^1^Psychiatric hospitals. ^2^Nursing home.

The patient was a single, unemployed, African American male with a psychiatric history of longstanding DSM-5 schizophrenia. He was treated with haloperidol decanoate and had a history of mixed substance abuse (including alcohol, cocaine, and* Cannabis*) but did not smoke tobacco. The patient had a family history positive for schizophrenia in a brother and paternal uncle. He graduated from high school. His history included multiple arrests and revolving door admissions (Figure 2).

His psychiatric treatment first started as an outpatient with haloperidol decanoate at 18 years of age. His first psychiatric admission to the first state psychiatric hospital was at the age of 27 and lasted almost 1 month. His second admission was at the age of 30 and lasted 6 days. He had no catatonic symptoms during these first two admissions.

The third hospitalization at the age of 30 was a result of medication noncompliance in the community. On day 1 of the third admission (used as day 1 of the long follow-up of this patient), his presenting symptoms included markedly disorganized thinking, bizarre behavior, poor self-care, social withdrawal, aggressiveness towards his brother, suspicion, and hallucinations leading to an involuntary admission per court order. During this admission, he was also diagnosed with obesity, hypertension, and dyslipidemia. The medical treatment included hydrochlorothiazide 25 mg/day on day 281. On day 438, two medications were added: simvastatin 20 mg/day and metoprolol 12.5 mg/day (increased to 100 mg/day on day 460).

During this third hospital admission, the patient's symptoms remained refractory to high doses of oral and decanoate haloperidol, olanzapine, and high doses of risperidone. Clozapine was started on day 190 at 25 mg/day and a stable dose of 400 mg/day was reached on day 228. Clozapine provided some improvement for the patient's symptoms. On day 392, a Mini-Mental Status Examination (MMSE) was administered to the patient with scores of 29/30 with WORLD backwards and 27/30 with serial 7s on the MMSE. This score is normal for his age and education [28]. On the MMSE, the abnormal results were serial 7s (3/5) and copying the two intersecting pentagons incorrectly. Brief Psychiatric Rating Scale-Anchored (BPRS-A) [29] provided a score of 37; abnormal items were severe suspiciousness, severe unusual thought content, moderately severe bizarre behavior, mild self-neglect, moderately severe disorientation, mild conceptual disorganization, moderately severe blunted affect, and moderate mannerisms and posturing. An Abnormal Involuntary Movement Scale (AIMS) exam [30] demonstrated some minimal movements in the tongue and lower 2 extremities and mild movements in the upper extremities.

#### 2.2.2. Clozapine Treatment in the First Year

He received an aripiprazole augmentation trial of clozapine from day 442 to day 532 up to 30 mg/day and of quetiapine up to 600 mg/day from day 532 to day 631. Then, the process of switching augmentation from quetiapine to ziprasidone was started on day 631.

### 2.3. First Catatonic Episode at 33 Years of Age during the Third Psychiatric Admission

#### 2.3.1. Clozapine Treatment Leading to the First Catatonic Episode

On day 659 when he was taking clozapine 400 mg/day (plus 200 mg/day of quetiapine and ziprasidone 120 mg/day), he ran away and had no access to any psychiatric (or medical) medication in the community.

During the prior 14 months when he was taking 400 mg/day of clozapine, his serum clozapine concentration was 511 ± 79 (range: 372–675), norclozapine concentration was 383 ± 80 (range: 194–507), and total clozapine concentration was 894 ± 129 (range: 566–1061).

#### 2.3.2. Catatonic Episode after the First Clozapine Withdrawal

On day 672, 14 days after running away from the psychiatric hospital and not receiving any other clozapine dose, he was arrested by the police and incarcerated; then, he was taken to the university hospital. He was diagnosed with dehydration, hypernatremia, hypochloremia, rhabdomyolysis, catatonic schizophrenia, hypertension, and hyperlipidemia. He had a normal head computerized tomography (CT) scan. The urine drug screen was positive only for clozapine; sodium was 150 mEq/L; chloride was 113 mEq/L; and he had an elevated CK of 1838 IU/L which decreased to 823 IU/L during admission. He was treated supportively with fluids. On day 673, he was discharged back to jail. Within 24 hours, he was found unresponsive and with urinary incontinence and was sent back to the university hospital on day 674. There, he was treated intermittently with doses of intravenous (IV) lorazepam 1 mg (we are unsure of the total dose) and quetiapine 25 mg/day. The patient refused food and drink during the entirety of the admission at the university hospital. On day 678, he was transferred back to the psychiatric state hospital where he presented limited interaction with his environment, poor eye contact, posturing of the left arm, stereotypic movement in the fingers, negativistic behavior, and bursts of dyskinetic movements. He was oppositional to any movements in all directions.

#### 2.3.3. Treatment with High Doses of Lorazepam during the First Catatonic Episode

On day 678, he arrived at the psychiatric hospital. He received 4 mg lorazepam intramuscularly (IM) and within two hours he began drinking and he was able to take some steps and sit in a chair. He was psychotic and disoriented to location. Three hours later, he received 4 mg lorazepam (a total of 8 mg for day 678).

The next day (day 679), he received a total of 14 mg of lorazepam with minimal results. He was able to eat; however, he had urinary incontinence and a paucity of speech. On day 680, the patient appeared worse and was started on lorazepam 16 mg/day. There was mild improvement in catatonic symptoms; he was able to say two sentences every 5 minutes and was able to stand up and walk with help. He was also able to eat unassisted. After day 681, lorazepam 16 mg/day was scheduled until day 698. He was slowly improving and at times became hostile and possibly psychotic. On day 683, ziprasidone 80 mg/day was started for these symptoms; however, it was discontinued the next day. Over the next week, the patient's catatonic symptoms improved but he only mumbled “OK” when spoken to.

Lorazepam was reduced to 8 mg/day within 1 month of his catatonic presentation (on day 700). The patient was tapered to 2 mg/day over the next few weeks; however, his symptoms exacerbated and he was kept on 4 mg/day for the next few months. Lorazepam was eventually switched to clonazepam, which was finally discontinued on day 1332.

#### 2.3.4. Time between the First and Second Catatonic Episodes

The patient remained catatonia-free during the next 2 years of his admission before he was discharged on day 1517. He was discharged on 400 mg/day of clozapine.

His fourth admission to the psychiatric hospital, 3 months after discharge, lasted 3 months. His discharge psychiatric medications included clozapine 600 mg/day, oral haloperidol 15 mg/day, 100 mg of haloperidol decanoate every 3 weeks, and benztropine 2 mg/day at night. His fifth admission to the psychiatric hospital was seven months later and lasted 2 months. His discharge medications did not include clozapine. His sixth admission to the psychiatric hospital was 7 months later and lasted 10 months. His discharge medications from the sixth admission included clozapine 600 mg/day and diphenhydramine 50 mg at night. In summary, during these 3 admissions, he did not have catatonic symptoms.

### 2.4. Second Catatonic Episode at 37 Years of Age (111.1 Kg) Leading to the 7th Psychiatric Admission

#### 2.4.1. Clozapine Treatment Leading to the Second Catatonic Episode

The patient was readmitted to the psychiatric hospital 18 days after the last discharge (6th admission) when the police brought him in after he was discovered sitting on the street for 8-9 hours that day.

#### 2.4.2. Second Catatonic Episode after Clozapine Withdrawal

On arrival at the psychiatric hospital on day 2509, the patient did not speak or move, he appeared to be responding to internal stimuli, he maintained poor eye contact, and he was malodorous and dirty. The patient had an elevated temperature (38.9°C), lower extremity edema, elevated heart rate and blood pressure, and a CK of 1943 IU/L.

#### 2.4.3. Treatment with High Doses of Lorazepam during the Second Catatonic Episode

He was sent to the emergency room at the university hospital the next day and was transferred to the intensive care unit where he was treated with IV fluids and IV antibiotics for a urinary tract infection. He was given 1 mg of lorazepam IV and then doses of 1 or 2 mg IV every 2 hours reaching up to 24 mg/day. He was also treated with a clonidine patch 0.1 mg/day and subcutaneous heparin 1500 units/day for preventing deep vein thrombosis. Head CT scan and echocardiogram were normal. After 5 days, he was returned to the psychiatric hospital after the CK was normalized.

On day 2514 when he came back to the psychiatric hospital, he was prescribed 22 mg/day IM lorazepam. The lorazepam taper took more than 3 months because there were 4 episodes suggestive of catatonic decompensation which required increasing the lorazepam dose again. Finally, on day 2649, lorazepam was completely and definitively stopped.

#### 2.4.4. Time between the Second and Third Catatonic Episodes

On day 2649, the same day lorazepam was discontinued, clozapine 50 mg/day was started. It was increased up to 400 mg/day on day 2720.

### 2.5. Third Catatonic Episode at the Age of 38 Years (132.3 Kg) Leading to the 8th Psychiatric Admission

#### 2.5.1. Clozapine Treatment Leading to the Third Catatonic Episode

On day 2903, his discharge medications from the seventh admission included clozapine 400 mg/day.

In the last 4 months, the five measured concentrations were 624 ± 69 (range: 504–684) for clozapine, 396 ± 97 (range: 253–520) for norclozapine, and 1020 ± 158 (range: 757–1167) for the total.

#### 2.5.2. Third Catatonic Episode after Clozapine Withdrawal

Six days after discharge, the patient was arrested and at that time he had the complete clozapine supply that he was given on discharge, indicating that he had not taken any clozapine tablets after leaving the hospital. He spent another 4 days in jail; then, after a total of ten days without clozapine, he was readmitted during his third catatonic episode for his 8th psychiatric admission. While in jail, the patient was described by law enforcement as not following directions or answering questions. He was brought to the psychiatric inpatient state hospital on the evening of day 2913 where his initial assessment described him as mute, not cooperative, displaying poor eye contact, and paying little attention to the interviewer. He had reduced psychomotor activity; he would sit very still; however, he would then abruptly start pacing around the room, and at one point he was pacing and then stopped and urinated. He also had some choreoathetoid movements in his fingers. This catatonic presentation was similar to the other 2 prior episodes.

#### 2.5.3. Treatment of the Third Catatonic Episode with Benzodiazepines and Clozapine

The charted information on the response to treatment and the justification for the treatment decisions during this third catatonic episode were much more limited than in prior episodes. During the third catatonic episode, there was a psychiatrist at the psychiatric hospital who started high doses of IM lorazepam but this treatment was tapered at the university hospital when the patient was admitted for serious dehydration.

In the first two days after coming from jail to the psychiatric hospital on day 2913, the patient refused to take oral medications and received IM cocktails with lorazepam 2 mg, diphenhydramine 50 mg, and haloperidol 5 mg. On day 2916, the patient was not eating or drinking, was mute, refused medications, and had periods of prolonged standing and staring, along with negativism. He received lorazepam 4 mg IM on that day. On the fourth day (day 2917), he received 12 mg of lorazepam IM and on the fifth day (day 2918) he received 16 mg of lorazepam IM. He showed clear signs of dehydration with blood urea nitrogen (BUN) of 32 mMol/L (range: 7–18), a sodium level of 157 mEq/L (range: 136–145), and CK elevation to 2307 U/L. Due to these lab abnormalities, on the sixth day (day 2919), the patient was sent back to the university hospital. His head CT scan was normal. He was diagnosed with catatonia and given IV fluids, and within 2 days his CK levels returned back to normal. The medical hospital discontinued all of his medications except oral lorazepam which was decreased to 3 mg/day.

He was transferred back a second time to the psychiatric hospital on day 2921. On that day, the patient continued to portray catatonic symptoms; he would sit still with his lunch on his lap and not eat even when encouraged by staff. Staff stated that he did not react to his external environment. He was prescribed no medication for 4 days. Six days later, he was prescribed 8 mg/day of lorazepam and 400 mg/day of clozapine. He had a third short admission to the university hospital for less than 2 days.

The patient appeared to improve from the catatonic symptoms due to the use of lorazepam, but it was used intermittently. Clozapine treatment was started first by adding it to lorazepam on day 2926. The patient took up to 400 mg/day intermittently until day 2941. During that time, he required frequent haloperidol and diphenhydramine injections, until a forced medication order was approved by the court. After that, a second trial of clozapine was started on day 2976 with 25 mg/day and slowly titrated up. His clozapine dose was slowly increased to 400 mg/day (on day 3079) and then to 600 mg/day (day 3140). This second clozapine trial led to a complete and definitive recovery from the catatonic episode.

#### 2.5.4. Stabilization and Final Placement

Shortly after that, the patient first collaborated with weight measurements, which indicated that he had lost 25 Kg during the catatonic episode. Before completing the third year of this 8th admission, on day 3603, at 41 years of age, the patient was transferred to a state nursing home for psychiatric patients. His discharge medications were clozapine 700 mg/day, metoprolol extended release 50 mg/day, simvastatin 20 mg/day, memantine 10 mg/day, and aspirin 81 mg/day. He stayed on clozapine until day 4084. On day 4129, he was transferred to another state psychiatric hospital located near the state nursing home. Since then, he has had another 4 admissions to this second state psychiatric hospital. The fifth admission started on day 4974 and has lasted more than 1 year. His current age is 44 years. Clozapine has never been prescribed during any of the 5 admissions to this second state psychiatric hospital.

## 3. Discussion

### 3.1. Assessments in Our Case

#### 3.1.1. Assessments by DSM-5 Catatonia Criteria

Each one of the three catatonic episodes was diagnosed at the time of presentation and retroactively by us through chart data using DSM-5 criteria (including negativism, mutism, and stupor). In all three episodes, CK levels were elevated and medical hospitalization and intravenous fluids were needed.

#### 3.1.2. Assessments by the MLADRCAT

All the authors agreed that the modified LADRCAT was definite in all three episodes indicating that the catatonic episodes were secondary to clozapine withdrawal. No other catatonic episodes were described from ages 17 to 42 years, indicating that the patient did not have any other cause of catatonia.

#### 3.1.3. Clozapine Dose and Serum Concentrations

The first and third catatonic episodes occurred after sudden withdrawal from 400 mg/day while the second was after withdrawal from 600 mg/day. In the first and second episodes, we have some idea of the patient's serum clozapine concentrations before the sudden withdrawal. As far as we can tell, the patient stopped clozapine suddenly after leaving the hospital (running away in the first episode or after prior discharge instances in the second and third episodes). An exact onset of the catatonic symptoms can only be approximated since the first and third catatonic episodes started in jail and in the second episode the patient was brought by the police after sitting for 8-9 hours on the street. At the hospital, the catatonic symptoms in the three episodes were definitely described between 10 and 18 days after sudden withdrawal of clozapine. After 1 week of sudden discontinuation of therapeutic serum clozapine concentrations, clozapine may disappear completely from the body [31].

### 3.2. Comparison of the Treatment of the Three Catatonic Episodes

During the first 2 episodes, the patient was treated for extended periods of time with high doses of lorazepam. In the first episode, he received 16 mg/day lorazepam for three weeks before significant improvements in his symptoms were documented and the dosage started to be tapered. During the second catatonic episode, he received up to 24 mg/day and slowly improved over a period of months due to multiple premature tapering attempts of lorazepam. In the third episode, the patient received 16 mg/day for only one day before being tapered to 8 mg/day. Clozapine up to 400 mg/day was prescribed but the patient was noncompliant with it. After a second and definitive clozapine trial during this third catatonic episode, the patient recovered completely from the catatonic symptoms. Due to medication noncompliance and lack of consistent treatment with lorazepam and/or clozapine, it took almost 3 months before the catatonic symptoms were resolved, after adding clozapine a second time.

Unlike other similar cases in the literature which required much lower lorazepam doses for resolution of symptoms, this patient required very high doses. The authors theorize that this patient may have required such high doses due to adaptation and desensitization of the benzodiazepine receptors due to the low dose exposure of lorazepam. In the first episode, the patient received 1 mg of lorazepam IV as needed at the university hospital for 4 days; the total dosage amount is uncertain. In the second episode, the patient responded to a high dose of 24 mg/day, prescribed after the last author's recommendation.

### 3.3. Comparison with Prior Published Cases

#### 3.3.1. Diagnosis of Catatonia

A literature review identified 9 other cases of clozapine-withdrawal catatonia (Tables 1 and 2). In 2 of the 9 cases, we were unable to make the catatonia diagnosis for the reduced number of symptoms described in the published articles [22, 23]. In the remaining 7 cases [15–21], the authors described ≥3 DSM-5 catatonia symptoms (Table 2).

#### 3.3.2. Diagnosis of Catatonic Episode Secondary to Clozapine Withdrawal

The MLADRCAT was applied to the 7 previously published cases of DSM-5-diagnosed catatonia. Three of the 7 cases were classified as “definite” catatonia after clozapine withdrawal and four as “probable” (Table 1). One of the 7 cases was reported to have included catatonia episodes independent of clozapine withdrawal. In the 7 previously published cases, the onset of catatonic symptoms after clozapine withdrawal ranged from 1.5 to 7 days versus 10–18 days in our patient (Table 1).

#### 3.3.3. Treatment of Catatonic Episode Secondary to Clozapine Withdrawal

In the 7 previously published cases, treatment varied including oral/IM lorazepam, clozapine, and ECT (Table 2). Resolution of symptoms in the literature ranged from 4 days to 3 weeks (Table 2). Our patient received high doses of lorazepam as a treatment for all 3 of his catatonic episodes; however, during his third episode, he only received a high lorazepam dose (16 mg) for 1 day before becoming dehydrated and requiring hospitalization. He was restarted on a lower dose of only 8 mg/day lorazepam with adjunct clozapine 400 mg/day taken intermittently due to noncompliance. He finally recovered from the third catatonic episode during a second and definitive clozapine trial.

#### 3.3.4. Underlying Psychiatric Disorder Treated with Clozapine

The psychiatric diagnosis in the 7 previously published cases included 5 with schizophrenia and 2 with schizoaffective disorder. Our patient was diagnosed with schizophrenia.

### 3.4. Pharmacological Mechanisms Which May Explain Catatonia after Sudden Clozapine Withdrawal

A pharmacological model of catatonia secondary to clozapine withdrawal is hypothesized after reviewing three pieces of literature data suggesting that clozapine may have agonist properties at the GABA receptors: (1) clozapine studies on GABA neurotransmission; (2) the existence of cases of a rare pharmacodynamic drug-drug interaction (DDI) when starting clozapine in patients taking benzodiazepines; and (3) two cases of clozapine intoxication who responded to flumazenil, a benzodiazepine antagonist at the GABA receptor.

#### 3.4.1. Clozapine Studies on GABA Neurotransmission

In a transcranial magnetic stimulation study, Kaster et al. [32] concluded that clozapine treatment is associated with an increase in GABA_B_ mediated inhibitory transmission. An in vitro study described clozapine as inhibiting GABA-evoked chloride currents at recombinant GABA_A_ receptors [33]. In a rat study, Bragina et al. [34] suggested that clozapine increases GABA activity by upregulating vesicular GABA transporters (VGAT) in the frontal cortex. In an animal study, Marx et al. [35] proposed a different mechanism that suggests that clozapine may indirectly increase GABA activity by releasing the neurosteroid, allopregnanolone. Finally, not all studies agree that clozapine has GABA agonist properties; in a 2000 in vitro study using a culture of ventral tegmental neurons, Michel and Trudeau proposed that in that model clozapine may have an inhibitory effect on GABA_A_ receptors [36].

#### 3.4.2. Clozapine and Benzodiazepine Pharmacodynamic DDI in Patients

A poorly understood pharmacodynamic DDI between clozapine and benzodiazepines has been described in a few individuals, usually leading to severe ADRs within 48 hours after the first clozapine dose in patients taking benzodiazepines. The ADRs secondary to this DDI may include lethargy, ataxia, loss of consciousness, and, rarely, respiratory arrest. Sassim and Grohmann [37] first reported two cases; then, they reviewed 959 patients taking clozapine and found 4 cases of severe cardiovascular and respiratory dysregulation during the combination of clozapine-benzodiazepines at the beginning of the clozapine titration [38]. Finkel and Schwimmer [39] reported 7 cases of respiratory arrest after treating 12,000 individuals, but only in 2 of these cases did the documentation associate them with the combination of benzodiazepines and the first clozapine doses. Klimke and Klieser [40] reported that, of 162 individuals treated with the combination of clozapine and benzodiazepines in a German hospital, 1 death by respiratory arrest occurred in an individual suffering from liver impairment. Faisal et al. [41] reviewed company data and found (1) 6 US cases of respiratory depression/arrest in combination clozapine-benzodiazepine treatment in 15,311 clozapine patients and (2) 10 European cases of cardiorespiratory arrest within the first 3 days (some taking benzodiazepines) in 63,000 patients. Bitter et al. [42] proposed that this DDI is rare. In conclusion, the occurrence of respiratory arrest during the coadministration of benzodiazepines and clozapine appears to be a very rare idiosyncratic reaction in the first days of clozapine treatment. The most reasonable explanation of this DDI is that clozapine and benzodiazepines may have additive properties at the GABA receptors which manifest in this way in rare individuals.

#### 3.4.3. Flumazenil Treatment of Drug Intoxication after Adding Clozapine to Low Benzodiazepine Doses

Peetoom and Schulte [43] published 2 cases in which flumazenil reversed comas secondary to adding clozapine to low doses of benzodiazepines. A 77-year-old female was taking other medications and 20 mg/day of oxazepam, but she went into a coma after erroneously taking 100 mg clozapine, 10 mg oxazepam, and 400 mg norfloxazine. She quickly recovered from the coma with 0.5 mg of flumazenil. A 44-year-old female with Huntington's disease was taking 6 mg/day of diazepam and 75 mg/day of oxazepam and other medications. She was titrated to 175 mg/day of clozapine within 14 days and went into a coma 2 hours after administration of her benzodiazepine medications. The patient awoke from the coma after the administration of 0.5 mg flumazenil [43]. That a coma induced by the combination of clozapine and benzodiazepines responded to flumazenil suggests that the coma may be explained by the additive effects of these drugs at the GABA receptors.

#### 3.4.4. GABA Mechanisms and Catatonia Secondary to Clozapine Withdrawal

According to the literature, dysregulation of the GABA receptors may contribute to catatonia. Hauser et al. [44] and Rosebush and Mazurek [45] described catatonic episodes during sudden benzodiazepine withdrawal. Carroll [46] described a patient with no previous history of catatonia who developed catatonia after abrupt withdrawals of benzodiazepines (GABA_A_ allosteric agonists) and baclofen (GABA_B_ agonist) [46]. In a review of catatonia after sedative withdrawal, Oldham and Desan [47] reviewed prior published cases and added new ones including 16 cases of benzodiazepines, 4 cases of benzodiazepines mixed with other sedative drugs, and 6 cases of alcohol withdrawal. Similar to the benzodiazepines, ethanol had also been described as an allosteric GABA_A_ agonist [48] and chronic alcoholism had been associated with GABA_A_ downregulation [47]. Oldham and Desan proposed that catatonia and delirium may be a spectrum of disorders associated with GABA receptor dysregulation [47].

In summary, if clozapine has GABA agonist properties, sudden clozapine withdrawal may explain GABA dysregulation with sudden decrease in GABA activity that may contribute to the development of catatonic symptoms in vulnerable patients. In that sense, based on prior cases and on our case, the treatment for catatonia secondary to clozapine can be benzodiazepines and/or restarting clozapine. In the USA, there is no parenteral clozapine; therefore, parenteral benzodiazepines may be required if the patient cannot take oral clozapine.

### 3.5. Limitations

The most important limitation of this case description is that the chart data is not detailed enough to know how catatonic symptoms responded to treatment in all 3 catatonic episodes. Ideally, one would have liked to have catatonia scales, standardized treatments not by multiple physicians, and exact descriptions of symptom onset before coming to medical attention. Even with its limitations, this is the first article with a careful description of 3 repeated episodes of catatonia secondary to sudden clozapine withdrawal and it includes a careful follow-up for decades (Figure 2), which is very rare in published case reports in psychiatry.

## 4. Conclusion

The LADRCAT was modified to assess ADRs secondary to drug withdrawal. This modified scale was used to assess 7 previously published cases of catatonia secondary to clozapine withdrawal and established that 3 were definitive and 4 probable. Another definitive case was described with three catatonic episodes which (1) occur after sudden discontinuation of clozapine in the context of decades of follow-up, (2) had ≥3 of 12 DSM-5 catatonic symptoms and serum CK elevation, and (3) required medical hospitalization and IV fluids. Clozapine may be a GABA agonist and sudden clozapine withdrawal may explain a sudden decrease in GABA activity that may contribute to the development of catatonic symptoms in vulnerable patients. Based on prior cases and on our case, the treatment for catatonia secondary to sudden clozapine withdrawal can be benzodiazepines and/or restarting clozapine.

## Supplementary Material

Twelve MLADRCAT figures: nine from previously published patients (Figures S1–S9) and three catatonic episodes of our patient (Figures S10–S12).

## Figures and Tables

**Table 1:** Published cases of catatonia associated with clozapine withdrawal identified by PubMed search^1^.

Authors	Country	Age	Sex	Dx	Clozapine		DSM-5 criteria for catatonia^3^	Modified Liverpool ADR Causality Category^4^
					Dose (mg/day)	Onset^2^ (days)		
Lee and Robertson [15]	New Zealand	30	M	S	350	1.5	Yes	Probable
Yeh et al. [16]	Taiwan	55	M	S	400	7	Yes	Definite
Bastiampillai et al. [17]	Australia	58	F	SA	300	3	Yes	Definite
Thanasan and Jambunathan [18]	Malaysia	ND^5^	M	SA	200	7	Yes	Probable
Wadekar and Syed [19]	USA	49	F	S	550	5	Yes^6^	Probable
Kumar et al. [20]	India	29	M	S	250	2	Yes	Probable
Wang et al. [21]	Australia	39	F	S	200	Immediate^7^	Yes	Definite
Shahrour et al. [22]	UAE	32	M	S	400	>28^8^	No^8^	Unlikely
Koychev et al. [23]	UK	22	M	PI	300	4	Unknown^9^	Unknown^9^

ADR: adverse drug reaction; DSM-5: Diagnostic and Statistical Manual of Mental Disorders, Fifth Edition; Dx: underlying psychiatric diagnosis; F: female; M: male; ND: not described; PI: psychotic illness; S: schizophrenia; SA: schizoaffective; UAE: United Arab Emirates; UK: United Kingdom; USA: United States of America.

^1^On 9/8/16, a PubMed search with the words “catatonia AND clozapine” was completed. We obtained 58 abstracts. All of them were reviewed for cases of catatonia after clozapine withdrawal. If the abstract or title appeared relevant, we obtained the article. After reviewing all articles, we identified these 9 cases of possible catatonia after clozapine withdrawal.

^2^Onset refers to the time period (measured in days) between sudden clozapine withdrawal and catatonia symptoms.

^3^The first and last authors determined whether a patient met Criterion A for DSM-5 catatonia (≥3 of 12 symptoms).

^4^The first and last authors developed the Modified Liverpool ADR Causality Assessment Tool to accommodate an ADR secondary to drug withdrawal. After reviewing each case and discussing it, the first and last authors selected a causality category by agreement. The flow chart of each case is available in the Supplementary Material.

^5^The patient is described as middle-aged.

^6^Catatonic symptoms were not described. The authors used the Bush-Francis scale in which 6/14 items on the screening instrument (a truncated version of the 23-item scale) were positive (≥2 is considered positive).

^7^The article described the patient as having been episodically noncompliant with clozapine. On four previous occasions in the immediate period following clozapine cessation, the patient developed florid psychotic symptoms in the form of persecutory, grandiose delusions, disorganized behavior, and auditory hallucinations. She displayed catatonic features (Bush-Francis scale score of 20, indicating severe catatonia) with excitement, mutism, posturing, staring, negativism, and echolalia.

^8^The patient was originally on clozapine and olanzapine. Both were stopped 4 weeks before symptoms. Then, he was restarted only on olanzapine and was discharged; 4 weeks later, he presented to the emergency room with symptoms. All the unusual symptoms described by the authors occurred after olanzapine withdrawal. The authors did not provide enough information for us to diagnose catatonia according to DSM-5 criteria; 1 of 12 DSM-5 catatonia symptoms was provided.

^9^We could not make a definitive judgment in this case. The article focused on an episode of clozapine withdrawal which was considered as potential neuroleptic malignant syndrome but the authors did not provide enough information for us to diagnose catatonia according to DSM-5 criteria. Another prior withdrawal episode after several days of clozapine cessation only described the patient's stupor, rigidity, and mutism, providing 2 of 12 DSM-5 catatonia symptoms.

**Table 2:** Details for 7 cases which were considered at least probable catatonic episodes secondary to clozapine withdrawal.

Authors	Catatonic symptoms	Treatment	Other catatonia episodes^1^
Lee and Robertson [15]	Restless, impulsive aggressive, disoriented disturbed sleep, refusal to wear clothing, staring, periodic posturing mannerisms, irrelevant speech, and uncontrollable laughing	Oral lorazepam 8 mg/day did not work Clozapine restarted 10 days after catatonia Symptoms resolved in 3 weeks	No

Yeh et al. [16]	Stupor, mutism, waxy flexibility, staring, posturing, negativism, and nil oral intake	Trihexyphenidyl 2–4 mg/day Clozapine restarted to 175 mg/day within 2 weeks Catatonia resolved in 7 days	One future

Bastiampillai et al. [17]	Urine/feces incontinence, mutism, mannerisms, stupor, posturing fever, diaphoresis, and autonomic instability	IM lorazepam unknown dosages Treated for 48 hours with no response ECT response started in the 4th session Major improvement in the 10th session	One past

Thanasan and Jambunathan [18]	Tremors in upper limbs, unresponsive to call or commands, febrile, flat affect, mute, and cogwheel and general rigidity	Bromocriptine from 2.5 to 15 mg/day (33 days) Oral diazepam for 6 days Improvement by day 12	No

Wadekar and Syed [19]	Fixed gaze, minimal withdrawal from pain, and Bush-Francis scale score 6/14	Lorazepam 1 mg IV with brief improvement Lorazepam 3 mg/day on days 1 and 2 Restarted on clozapine 25 mg/day on day 2 On 200 mg/day of clozapine by day 5 Rapid improvement on day 4	No

Kumar et al. [20]	Nil oral intake, quiet, mutism, posturing, and waxy flexibility	Lorazepam for 2 days ECT started on day 3 and improvement after 4 ECTs Discharged on risperidone 4 mg/day	No

Wang et al. [21]	Excitement, mutism, posturing, staring, negativism, and echolalia^2^	Inpatient admission with clozapine reinstitution	Four on clozapine withdrawal and five on lorazepam withdrawal^3^

ECT: electroconvulsive therapy; IM: intramuscular; IV: intravenous.

^1^Other catatonic episodes after clozapine withdrawal in the past or the future.

^2^On four previous occasions in the immediate period following clozapine cessation, the patient developed florid psychotic symptoms in the form of persecutory, grandiose delusions, disorganized behavior, and auditory hallucinations. She displayed catatonic features (Bush-Francis scale of 20 indicating severe catatonia).

^3^The patient initially had 4 episodes of catatonia after clozapine withdrawal. Then, she had 5 episodes of catatonia after lorazepam withdrawal, which were the focus of the article.
