# Supplementary material for: Catatonia Secondary to Sudden Clozapine Withdrawal: A Case with Three Repeated Episodes and a Literature Review
Source: Case Rep Psychiatry. 2017 Mar 15;2017:2402731. doi: 10.1155/2017/2402731 (PMC5370482; doi:10.1155/2017/2402731)
Supplement: Supplementary file 1 — Twelve MLADRCAT figures: nine from previously published patients (Figures S1–S9) and three catatonic episodes of our patient (Figures S10–S12). [file 2402731.f1.pdf]

Supplementary Material including
The Modified Liverpool Adverse Drug Reaction Causality Scale
Represented as Figures
from 9 Previously Published Cases (Figures S1-S9)
and 3 Catatonic Episodes from Our Patient (Figures S10-S12)

Case Reports in Psychiatry 2017

Title: Catatonia Secondary to Sudden Clozapine Withdrawal:

A Case with Three Repeated Episodes and

a Literature Review

Authors: John Bilbily, Betsy McCollum and Jose de Leon

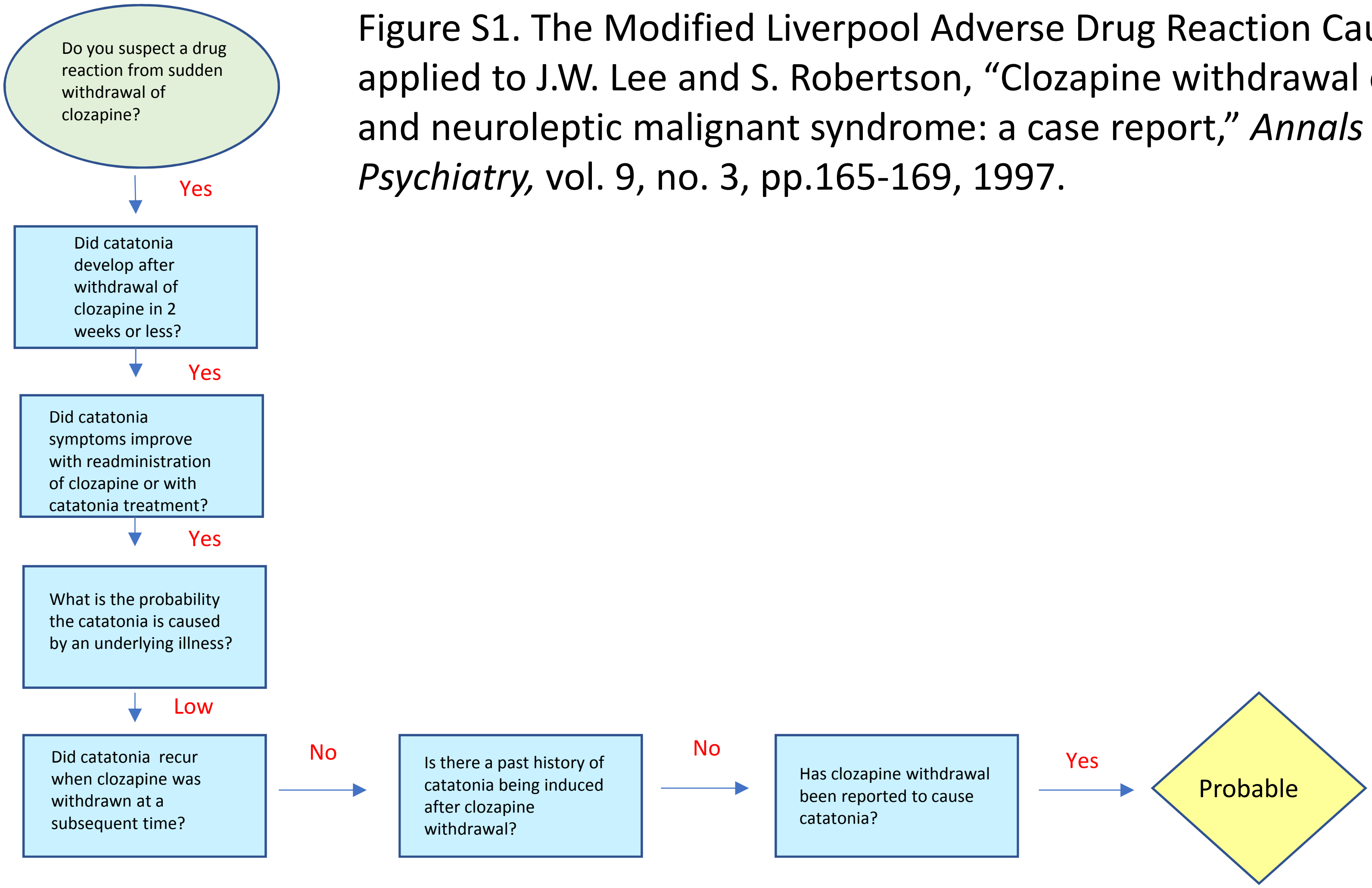

Figure S1. The Modified Liverpool Adverse Drug Reaction Causality Scale applied to J.W. Lee and S. Robertson, “Clozapine withdrawal catatonia and neuroleptic malignant syndrome: a case report,” *Annals of Clinical Psychiatry,* vol. 9, no. 3, pp.165-169, 1997.

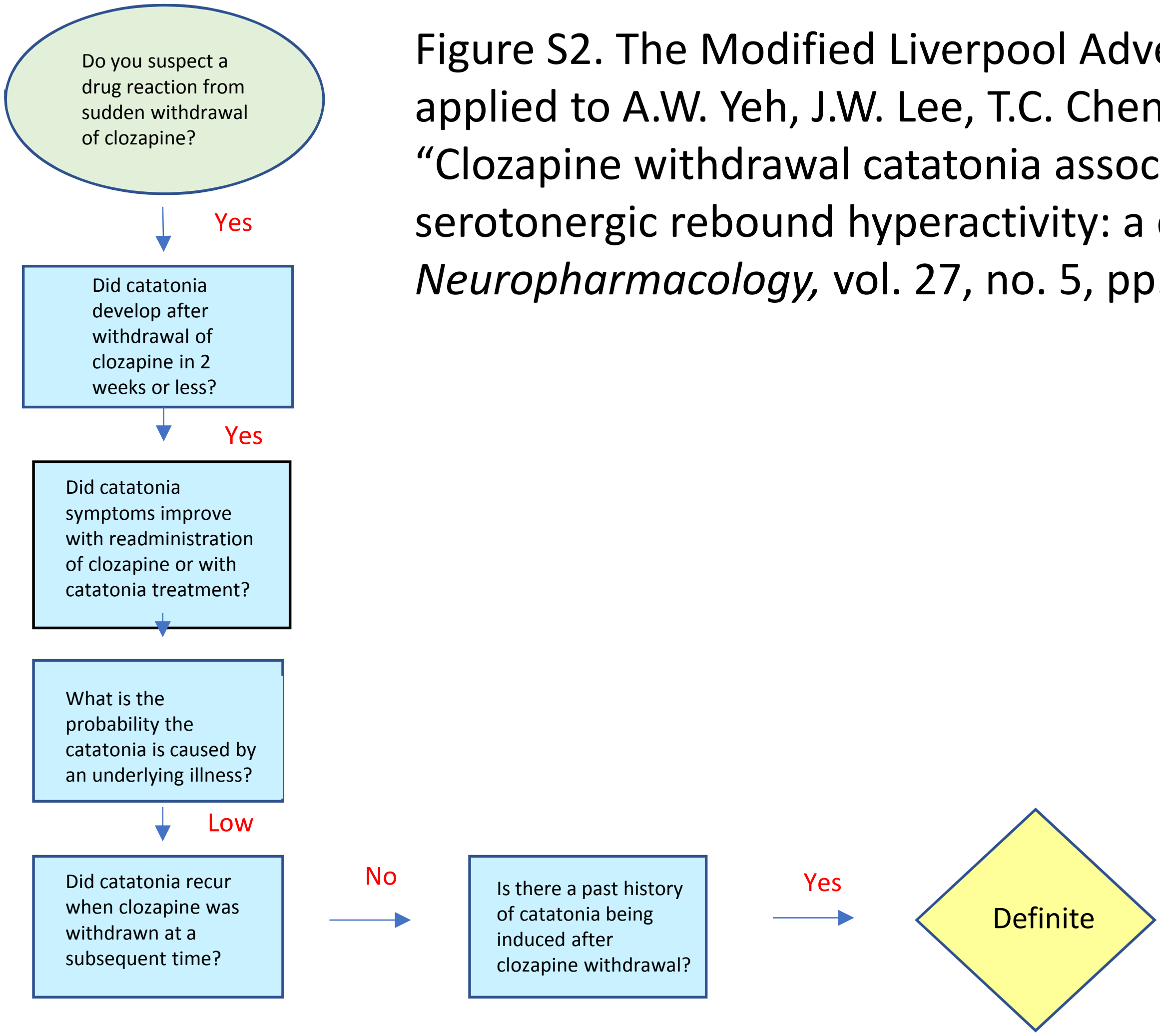

Figure S2. The Modified Liverpool Adverse Drug Reaction Causality Scale applied to A.W. Yeh, J.W. Lee, T.C. Cheng, J.K. Wen and W.H. Chen, “Clozapine withdrawal catatonia associated with cholinergic and serotonergic rebound hyperactivity: a case report,” *Clinical Neuropharmacology,* vol. 27, no. 5, pp. 216-218, 2004.

Figure S3. The Modified Liverpool Adverse Drug Reaction Causality Scale applied to T. Bastiampillai, F. Forooziya and R. Dhillon, “Clozapine-withdrawal catatonia,” *Australian & New Zealand Journal of Psychiatry,* vol. 43, no.3, pp. 283-284, 2009.

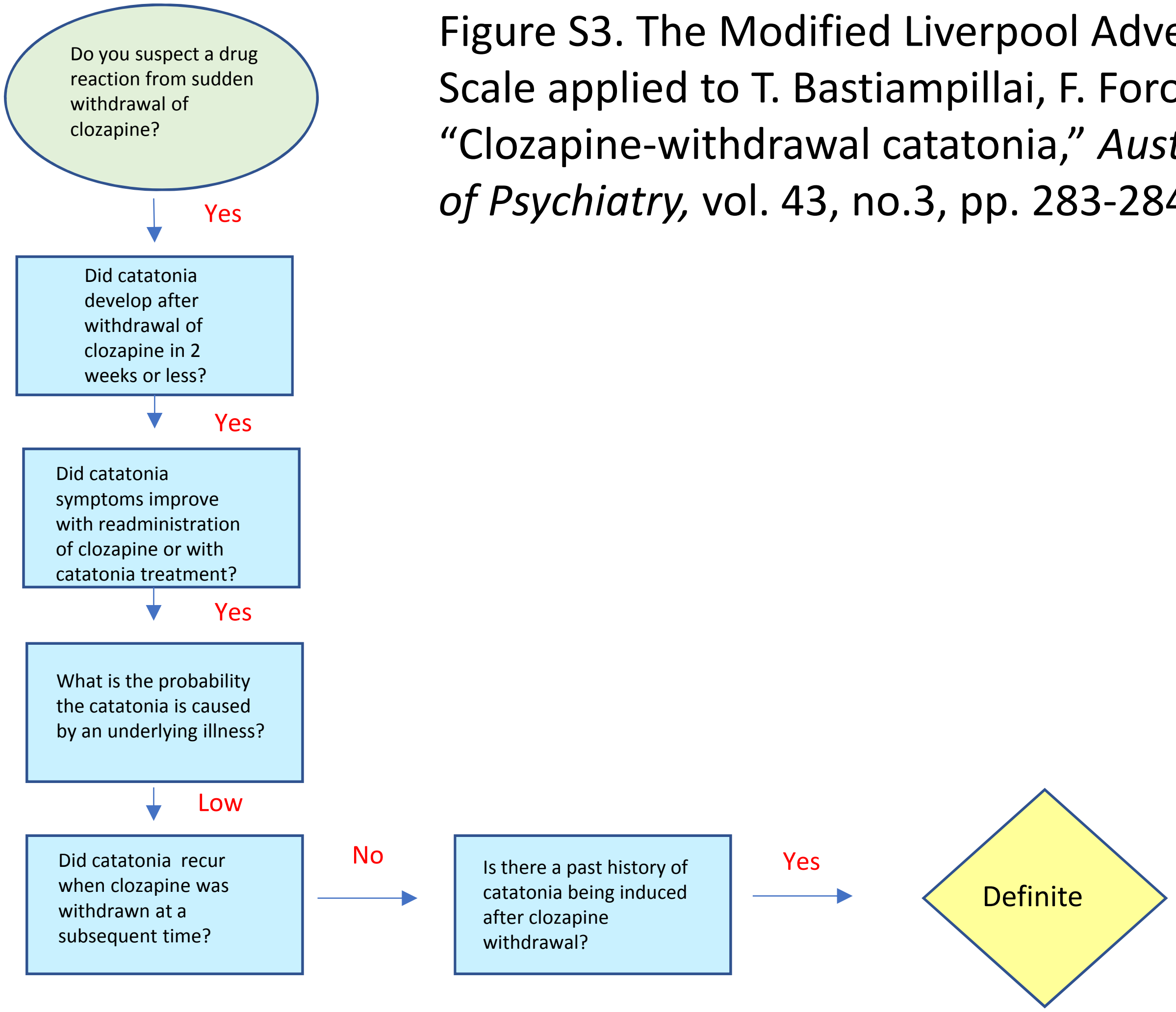

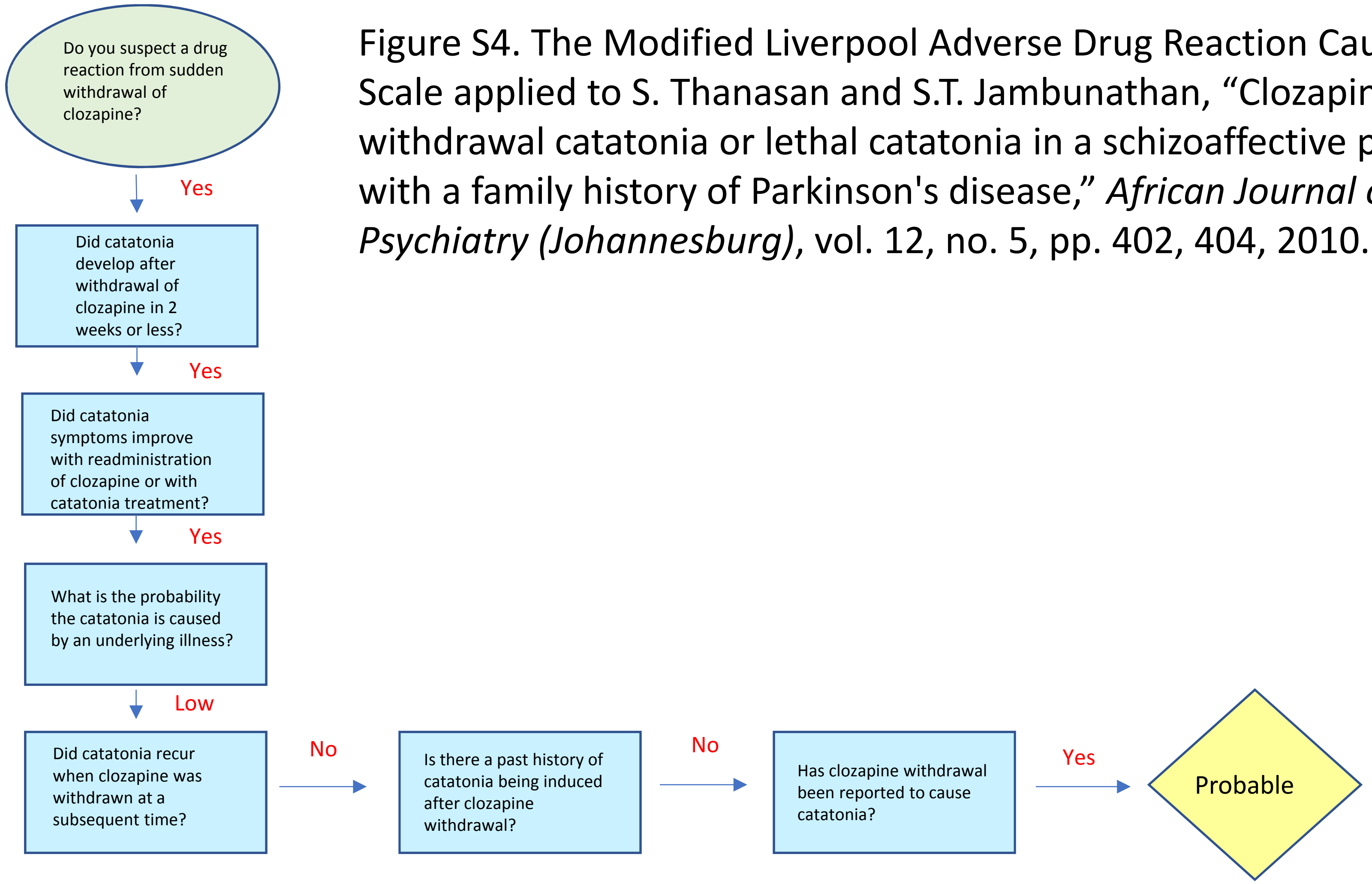

Figure S4. The Modified Liverpool Adverse Drug Reaction Causality Scale applied to S. Thanasan and S.T. Jambunathan, “Clozapine withdrawal catatonia or lethal catatonia in a schizoaffective patient with a family history of Parkinson's disease,” *African Journal of Psychiatry (Johannesburg)*, vol. 12, no. 5, pp. 402, 404, 2010.

Figure S5. The Modified Liverpool Adverse Drug Reaction Causality Scale applied to M. Wadekar and S. Syed, “Clozapine-withdrawal catatonia,” *Psychosomatics,* vol. 51, no. 4, pp. 355, 2010.

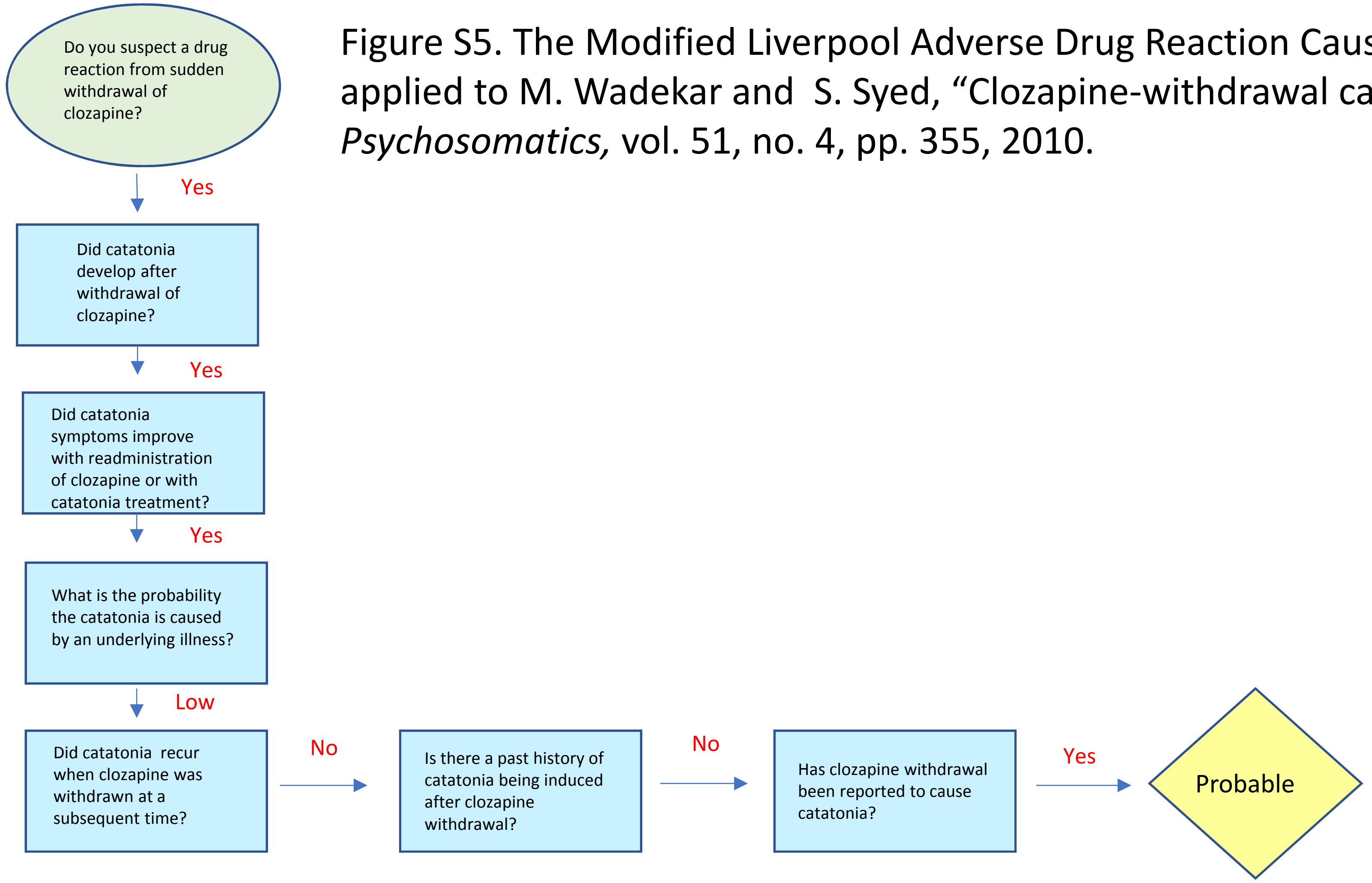

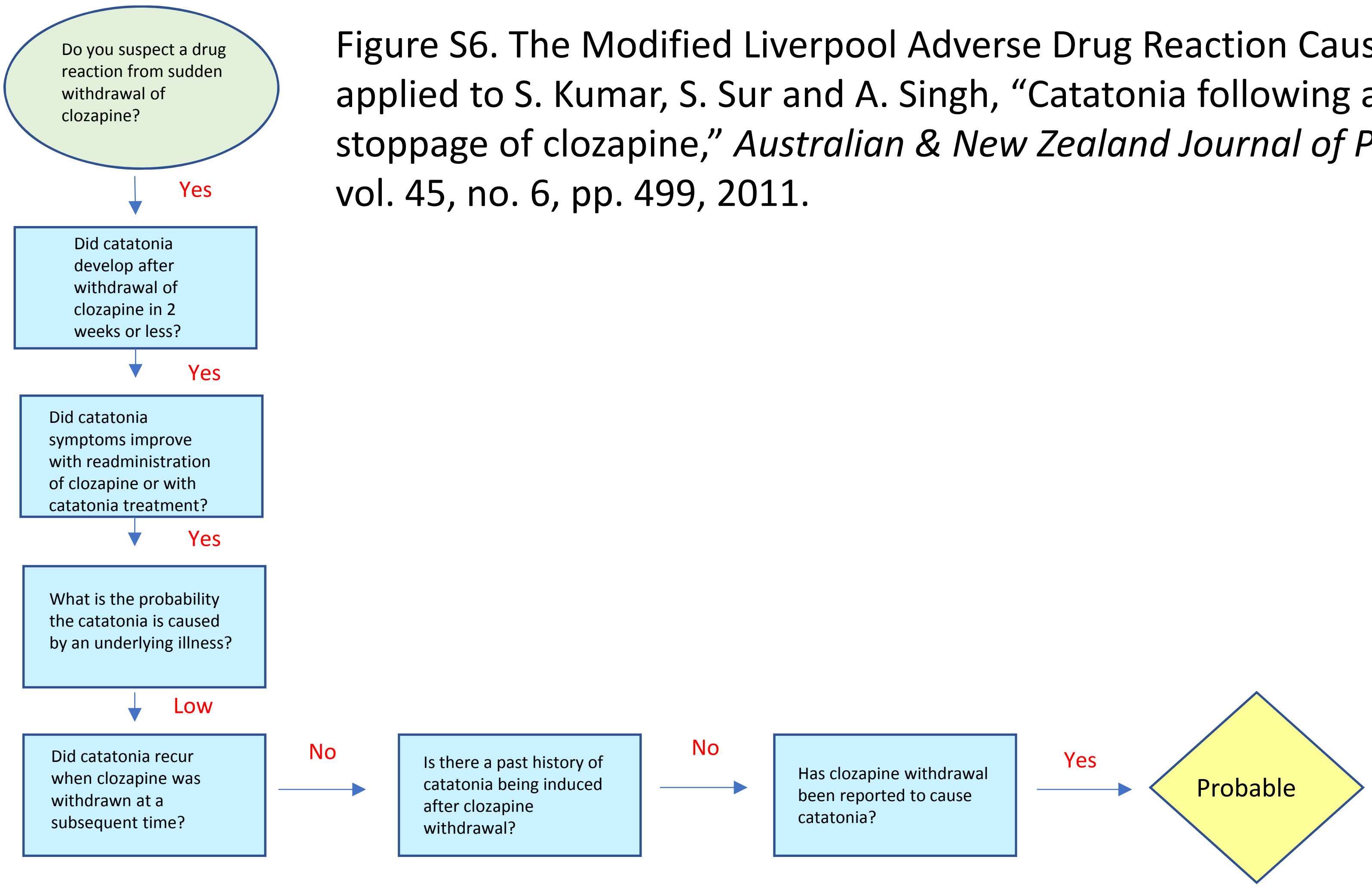

Figure S6. The Modified Liverpool Adverse Drug Reaction Causality Scale applied to S. Kumar, S. Sur and A. Singh, "Catatonia following abrupt stoppage of clozapine," *Australian & New Zealand Journal of Psychiatry*, vol. 45, no. 6, pp. 499, 2011.

Figure S7. The Modified Liverpool Adverse Drug Reaction Causality Scale applied to BZ. Wang, A. Gupta, T. Bastiampillai and F. Sani, “Recurrent clozapine and lorazepam withdrawal psychosis with catatonia,” *Australian and New Zealand Journal of Psychiatry*, vol. 46, no. 8, pp. 795-796, 2012.

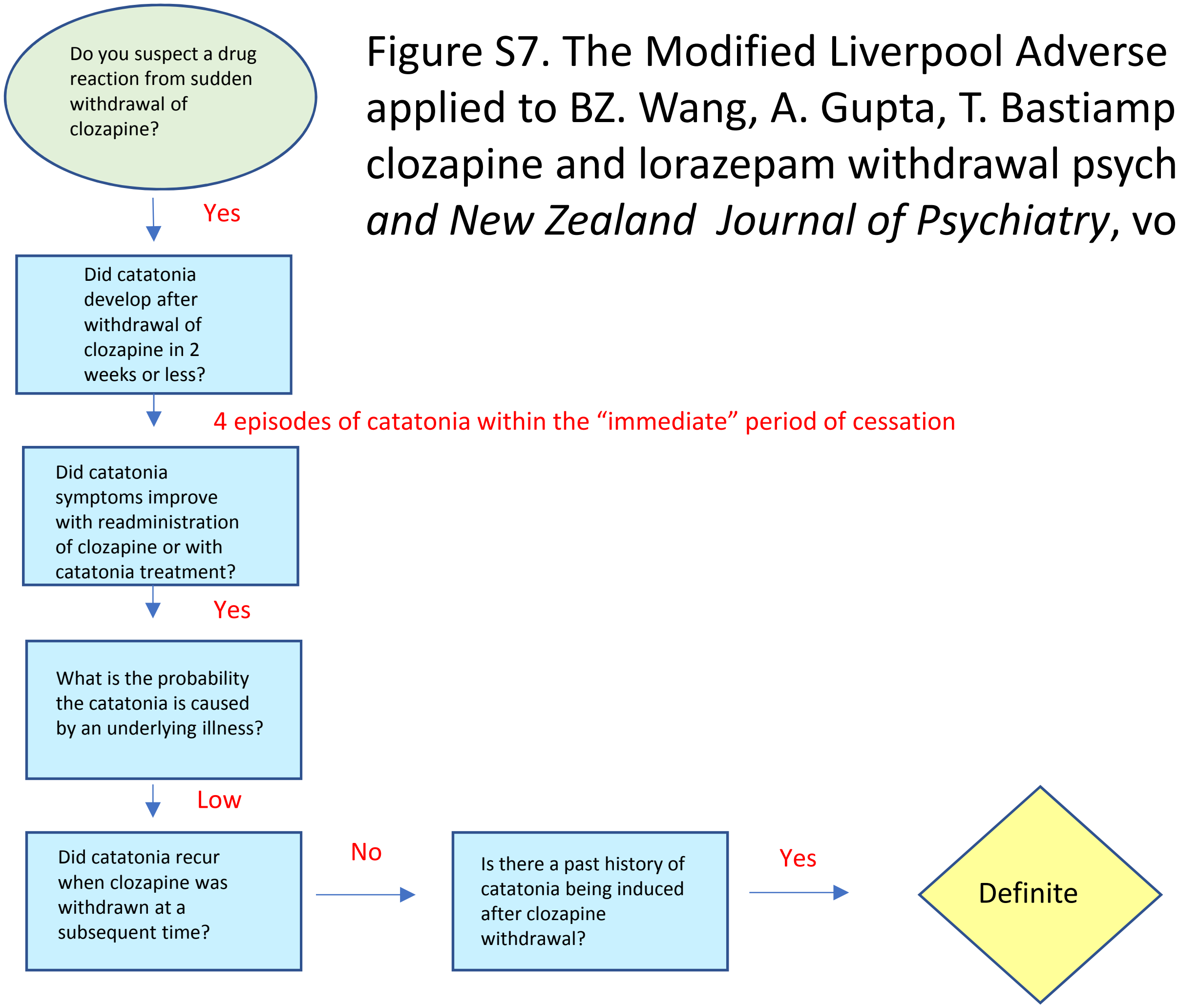

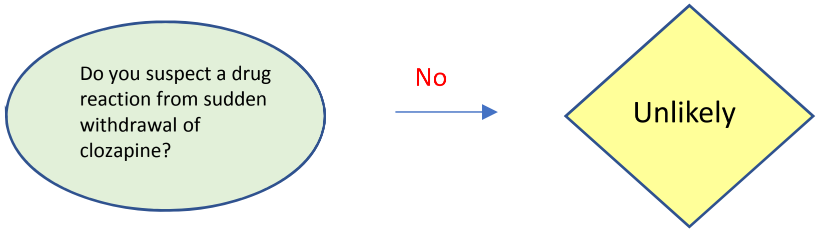

Figure S8. The Modified Liverpool Adverse Drug Reaction Causality Scale applied to T. Shahrour, M. Siddiq, S. Ghalib and T. Alsaadi, “Severe relapsing clozapine-withdrawal catatonia,” *Case Report in Psychiatry,* vol. 2015, no. 606853, pp.1-2, 2015.

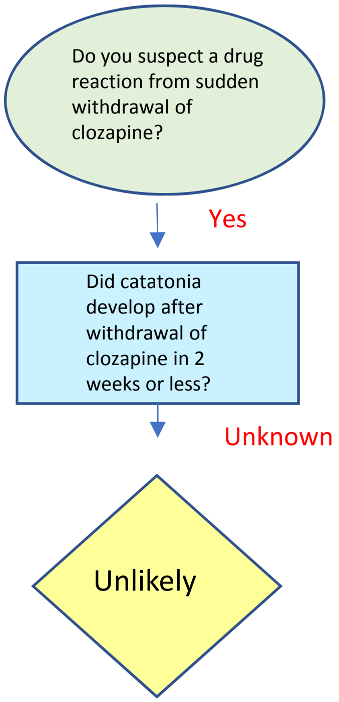

Figure S9. The Modified Liverpool Adverse Drug Reaction Causality Scale applied to I. Koychev, S. Hadjiphilippou, J. Lynch, P. Whelan and J. MacCabe, “Sudden-onset catatonia following clozapine withdrawal: a case report,” *Journal Clinical Psychiatry*, vol. 77, no.7, pp. e899, 2016.

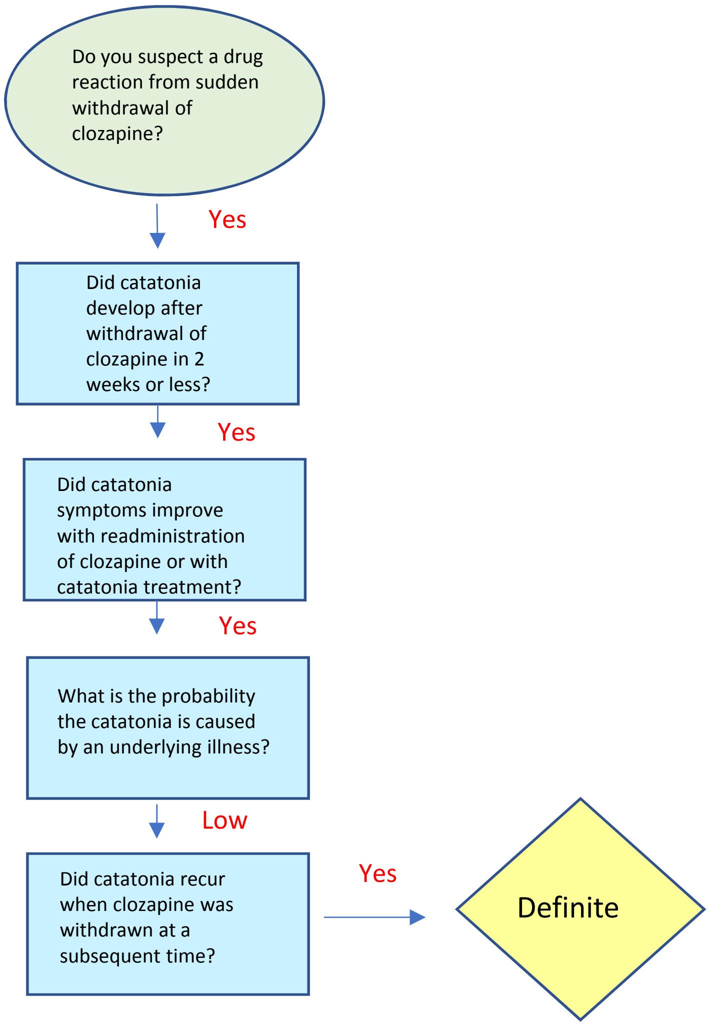

Figure S10. The Modified Liverpool Adverse Drug Reaction Causality Scale applied to our patient's 1st episode of catatonia secondary to clozapine withdrawal

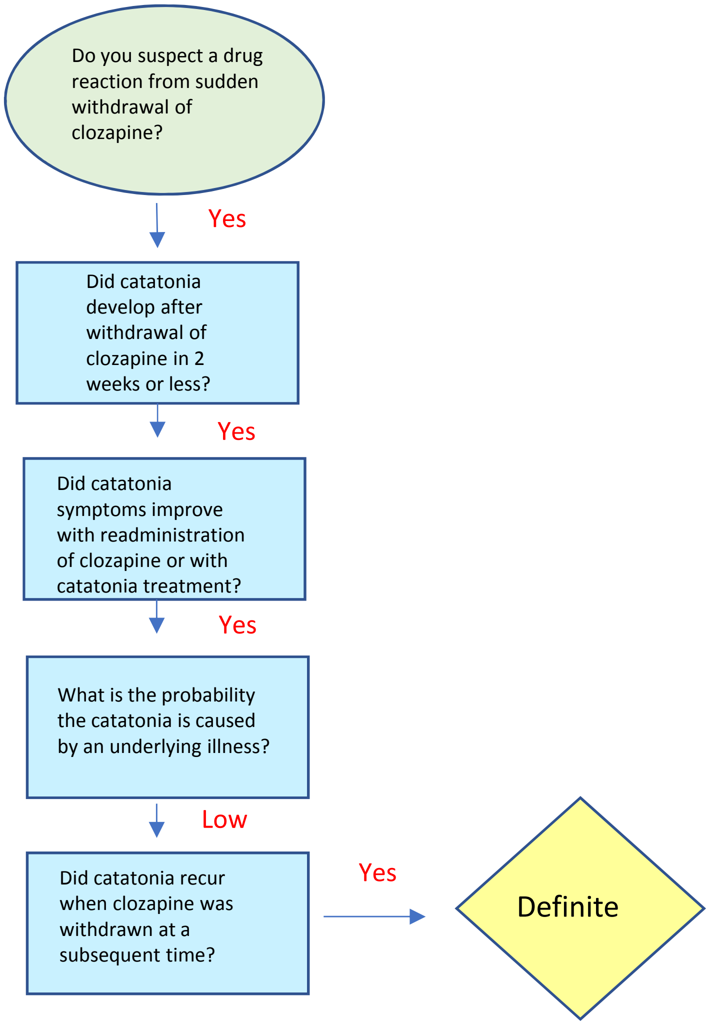

Figure S11. The Modified Liverpool Adverse Drug Reaction Causality Scale applied to our patient's 2nd episode of catatonia secondary to clozapine withdrawal

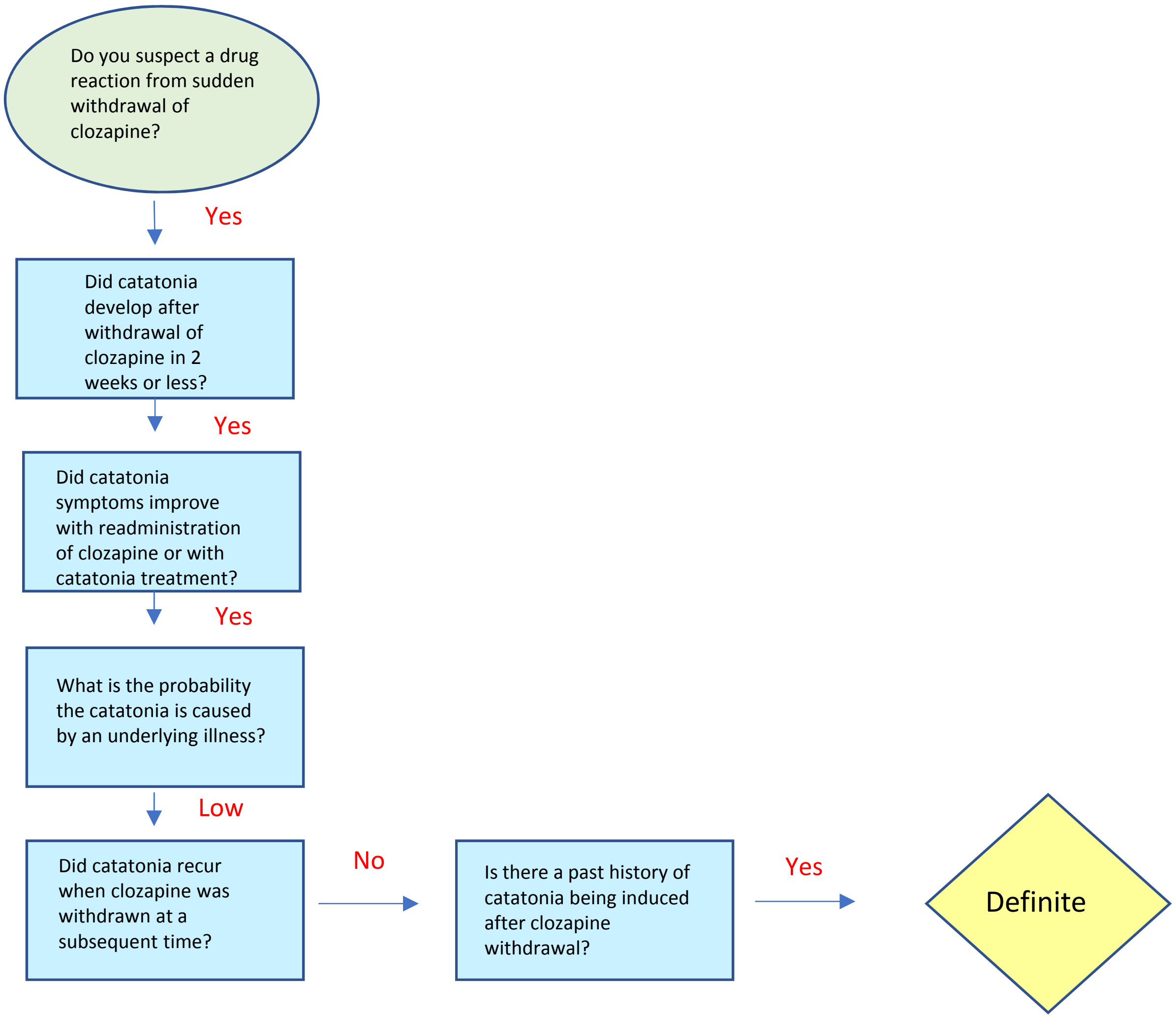

Figure S12. The Modified Liverpool Adverse Drug Reaction Causality Scale applied to our patient's 3rd episode of catatonia secondary to clozapine withdrawal
